# Pancreatic Stone Protein as a Versatile Biomarker: Current Evidence and Clinical Applications

**DOI:** 10.3390/diseases13080240

**Published:** 2025-07-31

**Authors:** Federica Arturi, Gabriele Melegari, Riccardo Mancano, Fabio Gazzotti, Elisabetta Bertellini, Alberto Barbieri

**Affiliations:** 1 School of Anaesthesia and Intensive Care, University of Modena and Reggio Emilia, 41121 Modena, Italy; federica.arturi.94@gmail.com (F.A.); mancano.ric@gmail.com (R.M.); alberto.barbieri@unimore.it (A.B.); 2 Department of Anaesthesia and Intensive Care, Azienda Ospedaliera Universitaria di Modena, 41125 Modena, Italy; gazzottifabio@gmail.com; 3 School of Medicine, University of Modena and Reggio Emilia, 41121 Modena, Italy; elisabettab53@gmail.com

**Keywords:** biomarker, sepsis, pancreatic stone protein

## Abstract

Background: The identification and clinical implementation of robust biomarkers are essential for improving diagnosis, prognosis, and treatment across a wide range of diseases. Pancreatic stone protein (PSP) has recently emerged as a promising candidate biomarker. Objective: This narrative review aims to provide an updated and comprehensive overview of the clinical applications of PSP in infectious, oncological, metabolic, and surgical contexts. Methods: We conducted a structured literature search using PubMed^®^, applying the SANRA framework for narrative reviews. Boolean operators were used to retrieve relevant studies on PSP in a wide range of clinical conditions, including sepsis, gastrointestinal cancers, diabetes, and ventilator-associated pneumonia. Results: PSP has shown strong diagnostic and prognostic potential in sepsis, where it may outperform traditional markers such as CRP and PCT. It has also demonstrated relevance in gastrointestinal cancers, type 1 and type 2 diabetes, and perioperative infections. PSP levels appear to rise earlier than other inflammatory markers and may be less affected by sterile inflammation. Conclusion: PSP represents a versatile and clinically valuable biomarker. Its integration into diagnostic protocols could enhance early detection and risk stratification in critical care and oncology settings. However, widespread adoption is currently limited by the availability of point-of-care assay platforms.

## 1. Introduction

One of the most recent and detailed definitions of a biomarker was elaborated by the FDA-NIH working group in 2021, which defines it as a measurable indicator of a normal or pathological biological process or the effectiveness of a pharmacological or interventional treatment. Biomarkers can be derived from histological, radiographic, molecular, or physiological sources [1]. A specific biomarker can guide therapeutic decisions only after being rigorously validated and approved, as pointed out by the FDA [2]. Califf also highlights the importance of distinguishing biomarkers from clinical outcome assessments (COAs), which instead measure patient-centered outcomes such as symptoms or survival [3]. Considering this, the identification and clinical validation of reliable biomarkers remains a crucial goal in modern medicine. Pancreatic stone protein (PSP), initially discovered in the setting of chronic calcifying pancreatitis, has recently gained increasing attention as a promising biomarker across various pathological conditions. Unlike traditional inflammatory markers such as C-reactive protein (CRP) or procalcitonin (PCT), PSP appears to reflect early immune activation and cellular stress even before clinical symptoms or organ dysfunction emerge. This characteristic positions PSP as a valuable tool for improving the timeliness and accuracy of diagnosis in time-sensitive conditions such as sepsis, postoperative infections, and ventilator-associated pneumonia. In addition to infectious diseases, PSP has shown relevance in oncological and metabolic disorders, including gastrointestinal cancers and diabetes, suggesting potential roles in risk stratification and disease monitoring. Given the rapid growth of the literature on PSP, a comprehensive and structured synthesis is needed to support clinicians and researchers in understanding its potential applications and limitations. This narrative review addresses that gap, summarizing the clinical uses of PSP across diverse medical contexts. To ensure methodological rigor, this review was conducted using the Scale for the Assessment of Narrative Review Articles (SANRA) framework. SANRA provides structured guidance on article justification, aim formulation, literature search, referencing, and scientific reasoning, thereby enhancing the quality and transparency of narrative reviews [4].

## 2. Materials and Methods

This narrative review was conducted following the principles outlined in the SANRA framework, a validated tool developed to improve the methodological quality and reporting standards of narrative reviews [4]. We specifically referred to the most recent version published in 2019, which provides structured guidance on the justification of article importance, definition of aims, literature search strategy, referencing, and scientific reasoning. These criteria were applied throughout the development of this manuscript to ensure rigor, transparency, and relevance for the intended clinical readership.

For the identification and selection of relevant literature, we used PubMed^®^ as the primary database, given its extensive indexing of peer-reviewed biomedical publications. The search strategy incorporated Boolean operators (and, or, not) to refine and combine search terms in a logical and inclusive manner, maximizing both sensitivity and specificity. The keywords employed in our search included the following: “Pancreatic Stone Protein”, “Pancreatitis”, “Renal Disease”, “Diabetes”, “Cancer”, “Gastrointestinal cancer”, “Infections”, “Pneumoniae”, “Sepsis”, “Burns”, and “Surgery”. These terms were selected to capture the full range of clinical contexts in which pancreatic stone protein (PSP) has been investigated either as a diagnostic or prognostic biomarker or where it holds pathophysiological relevance. We applied no date restrictions in order to retrieve both recent and foundational studies. After the initial search, titles and abstracts were manually screened to identify articles aligned with the aim of the review. Subsequently, full texts were assessed to confirm eligibility. Only articles published in English and appearing in peer-reviewed journals were included in the final synthesis. Although this is a narrative review and not a systematic meta-analysis, we adopted a structured data extraction grid to classify the studies by clinical context, biomarker role (diagnostic vs. prognostic), comparison with standard markers (e.g., CRP, PCT), and reported outcomes. When available, we considered reported statistical metrics such as the area under the receiver operating characteristic curve (AUC), sensitivity, specificity, and predictive values to qualitatively assess the strength of evidence. Sensitivity (true positive rate) and specificity (true negative rate) were considered in relation to their ability to minimize false negatives (FNs) and false positives (FPs), respectively. AUC was used to quantify the global diagnostic performance of PSP, with values closer to 1.0 indicating excellent discrimination between disease and non-disease states. These parameters were evaluated to estimate the reliability, accuracy, and clinical applicability of PSP across various clinical conditions. This approach allowed us to preserve the narrative nature of the review while incorporating a semi-quantitative assessment of the available evidence.

## 3. Results: The Discovery of Pancreatic Stone Protein

The discovery of the pancreatic stone protein (PSP, also known as regenerating islet-derived protein 1-alpha (REG1A), is a secretory protein encoded by the gene UniProt ID: P05451), (Biomolecular Structure and Functional Profile of pancreatic stone protein (PSP) in Appendix A) and it has been proposed as a biomarker in various clinical settings. It dates back to the 1970s, from numerous gastroenterological studies on chronic calcifying pancreatitis (CCP). This medical condition has been described as caused by the formation of stones in the pancreatic ducts, brought about via an alteration in the protein composition of pancreatic juice, resulting in its precipitation. This alteration seems to be connected to alcohol abuse [5]. Subsequently, Guy et al. analyzed the chemical composition of stones in patients with CCP, identifying a high prevalence of high-molecular-weight protein. As a result, they hypothesized the central role of these proteins in the formation of calcium carbonate stones, which are considered the main factor responsible for the onset of CCP [6]. De Caro et al. have more accurately analyzed the chemical composition of pancreatic juice of both healthy subjects and those with CCP, confirming the conclusions of previous studies but also identifying another soluble, low-molecular-weight protein. This protein appears to be abundant in the pancreatic juice of healthy subjects but particularly low in subjects with the disease, allowing them to hypothesize a protective role of this protein against stone formation. This protein was identified as pancreatic stone protein [7]. Then, they demonstrated, through in vitro experiments, the PSP’s ability to inhibit the formation of calcium carbonate stones [8], results later confirmed in vivo, showing that normal levels of the protein prevented the formation of stones [9]. On the contrary, researchers proved very low levels of PSP in the pancreatic juice of patients developing CCP, confirming that low levels of the protein represent a key factor in the genesis of the clinical condition [10]. Over the decades, the role of PSP has been demonstrated in numerous pathological conditions, which we will analyze in detail in the following paragraphs [11,12]. (A detailed biomolecular description of PSP, including the gene name, isoforms, expression profile, and functional domains, is provided in Appendix A).

### 3.1. Significance of Pancreatic Stone Protein as a Biomarker

Pancreatic stone protein (PSP) has emerged as a novel biomarker with potential clinical utility across multiple medical domains. Originally identified in pancreatic secretions and associated with stone formation in chronic pancreatitis, PSP is now recognized for its role in systemic inflammatory responses. Its production increases in response to infection and physiological stress, particularly in critically ill patients. Unlike hepatic acute-phase reactants such as CRP and PCT, PSP is produced primarily in the pancreas and associated mucosal tissues, suggesting a different regulatory pathway and potentially earlier release during pathophysiological insults. Several studies have demonstrated that PSP rises earlier than CRP or PCT in the onset of sepsis, and its levels correlate with disease severity and mortality. Furthermore, PSP appears to be less affected by non-infectious inflammation, improving its specificity in distinguishing infectious from sterile inflammatory states. These characteristics suggest that PSP may be especially valuable in time-critical settings such as intensive care, perioperative care, and emergency medicine. However, broader adoption remains limited due to the availability of standardized assays and the need for further validation in large, multicenter cohorts [13,14].

### 3.2. PSP as a Diagnostic Biomarker in Sepsis

Sepsis is a potentially life-threatening condition caused by a dysregulated host response to infection, resulting in organ dysfunction, which distinguishes it from a simple infection. Septic shock is considered an advanced form of sepsis, characterized by profound cellular, metabolic, and cardiovascular abnormalities, and it is associated with substantially increased mortality. In this context, PSP has been studied with two main objectives: as a diagnostic and a prognostic biomarker in patients with sepsis and septic shock.

A primary focus of the literature has been the ability of pancreatic stone protein (PSP) to discriminate between non-infectious conditions and the presence of infection or sepsis. Eggimann et al. were among the first to emphasize the value of PSP as a biomarker for the early diagnosis of sepsis, demonstrating that its levels rise significantly even before clinical symptoms become apparent, correlating with both disease severity and organ dysfunction [14]. These findings were further confirmed and expanded by Prazak et al., who evaluated PSP outside the ICU setting and demonstrated its diagnostic utility across a broader hospitalized patient population with infectious diseases. Their results also suggested a potential role for PSP in guiding antimicrobial stewardship [15].

Comparative studies between PSP and traditional sepsis biomarkers such as C-reactive protein (CRP) and procalcitonin (PCT) have also been published. Pugin et al. showed that all three biomarkers increased before the clinical manifestation of sepsis, but PSP exhibited an earlier rise up to five days before symptoms compared to the 2–3-day elevation seen with CRP and PCT. Moreover, the combination of PSP with CRP and PCT improved diagnostic accuracy relative to each marker alone [16]. These observations were corroborated by Permana et al., who confirmed the early diagnostic role of PSP and emphasized its utility, alone or in combination, in initiating early treatment among high-risk patients, even before symptom onset, thereby potentially improving outcomes [17].

Numerous studies have also explored PSP’s prognostic value in sepsis, as summarized in Table 1. In preclinical models, Hu et al. demonstrated that PSP administration worsened sepsis severity in a dose-dependent manner, with higher levels associated with increased mortality [18]. To explain this finding, Ventura et al. proposed that PSP—being produced via the pancreas and not the liver—should not be considered a classic acute-phase protein. Rather, they highlighted its role in modulating innate immune responses, particularly by influencing polymorphonuclear neutrophil (PMN) activity [19].

Among human studies, Que et al. reported significantly higher PSP levels in patients with septic shock compared to those with sepsis and in both groups compared to non-infected individuals. PSP also emerged as the only biomarker—among CRP and PCT—that consistently predicted in-hospital mortality, with rising levels reflecting a worse prognosis [20]. A subsequent study by the same group demonstrated that combining PSP with CRP and PCT further improved predictive accuracy for mortality in ICU patients with septic shock [21].

Finally, Eggimann et al. reviewed the major advantages of PSP in the clinical management of sepsis, highlighting its utility in early diagnosis, risk stratification, and outcome prediction—key aspects in the treatment of critically ill patients [14]. From a health-economic perspective, Schneider et al. showed that incorporating PSP into diagnostic pathways can contribute to substantial cost savings by facilitating the more timely and accurate identification and management of sepsis and septic shock—both highly prevalent and resource-intensive conditions [22].

### 3.3. PSP in Pediatric Patients

The role of pancreatic stone protein (PSP) has gained increasing attention in the pediatric population, particularly in relation to infections and sepsis—conditions that remain major causes of morbidity and mortality in children. The most frequent underlying causes include respiratory tract infections, catheter-related bloodstream infections, hematologic malignancies, neurological disorders, and primary immunodeficiencies [23]. Clinically, pediatric sepsis often presents not only with signs of infection but also with systemic manifestations such as hemodynamic instability (e.g., hypotension, tachycardia), altered mental status, and dysregulated temperature control [24].

One of the earliest studies to evaluate PSP in this population was conducted by Saleh et al., who assessed its performance alongside apolipoprotein A-V and copeptin. Their results showed that PSP levels were significantly elevated in children with severe disease and higher mortality risk, suggesting its potential as a prognostic biomarker. Additionally, PSP levels were higher in septic children than in non-infected controls, supporting its diagnostic value as well [25].

Subsequent findings from a prospective cohort study by Bottari et al., involving 100 pediatric patients admitted to high-dependency care, confirmed the utility of PSP in early sepsis identification. The study proposed a diagnostic cut-off value of 167 ng/mL, which effectively distinguished between septic and non-septic patients. These results support the use of PSP as an aid in early diagnosis and targeted clinical management [26].

More recently, Dündar et al. reinforced PSP’s relevance in the pediatric intensive care setting by demonstrating its value in both diagnosis and mortality risk stratification among critically ill children. Finally, Antari et al. explored PSP in pediatric patients with hematologic malignancies who developed febrile neutropenia, a severe and potentially life-threatening complication. Their data revealed that PSP measurements allowed for the early detection of infection, facilitating the timely initiation of targeted therapy, which is essential to improving outcomes in this particularly vulnerable cohort [27,28].

### 3.4. PSP in Burns Patients

A particularly promising clinical application of pancreatic stone protein (PSP) is its use in the management of patients with severe burns. Klein et al. were among the first to underscore the urgent need for biomarkers capable of distinguishing between sterile inflammatory responses induced via thermal injury and those stemming from infectious complications. Conventional markers such as procalcitonin (PCT) and C-reactive protein (CRP), both of which are upregulated during systemic inflammation, often rise after tissue damage from burns or subsequent surgical procedures (e.g., debridement, grafting), thus limiting their specificity in this context. In contrast, PSP levels remain relatively stable in response to non-infectious inflammatory stimuli, making it a more reliable marker for differentiating infection from trauma-related inflammation in burn patients [29].

These observations have been corroborated via subsequent studies, which demonstrated that PSP levels increase significantly in response to infection, often up to 72 h before the onset of clinical symptoms. This temporal advantage offers clinicians a critical diagnostic window to initiate timely interventions, potentially improving outcomes and reducing associated healthcare costs [30].

Furthermore, the utility of PSP has been evaluated in burn patients who have sustained inhalation injuries, which primarily affect the upper airway and can result in systemic toxicity from agents such as carbon monoxide and cyanide. These injuries elicit widespread inflammatory responses marked by cytokine release and oxidative stress [31]. Notably, Klein et al. found that PSP levels are not significantly influenced by inhalation trauma, unlike CRP and PCT, thereby preserving its diagnostic accuracy for sepsis even in this complex clinical setting [32].

### 3.5. PSP in COVID-19 Infection

Since late 2019, the COVID-19 pandemic has posed a major global health challenge. The SARS-CoV-2 virus can cause severe respiratory failure and trigger a dysregulated inflammatory response that may progress to multi-organ dysfunction and increased mortality risk [33,34]. In this clinical context, the availability of reliable biomarkers for early diagnosis, disease monitoring, and prognostic assessment is essential. Several studies have investigated the role of pancreatic stone protein (PSP) in COVID-19. Melegari et al. reported a significant increase in serum PSP levels in COVID-19 patients, which paralleled elevations in other inflammatory markers such as C-reactive protein (CRP) and procalcitonin (PCT). Notably, higher PSP concentrations were associated with increased mortality [35]. Similarly, Van Singer et al. confirmed these findings, proposing PSP as a valuable biomarker for prognostic evaluation in patients with COVID-19 [35,36]. However, contrasting results were reported by Lagadinou et al., who found no significant association between PSP levels and mortality. Instead, they observed that elevated PSP values correlated with longer hospital stays, suggesting a potential role for PSP in predicting disease duration, rather than mortality [37]. These conflicting findings underscore the need for further research to clarify the diagnostic and prognostic utility of PSP in the context of COVID-19.

### 3.6. PSP in Ventilator-Associated Pneumonia (VAP)

Numerous studies have investigated the role of pancreatic stone protein (PSP) in infectious diseases affecting various organ systems, particularly within the respiratory tract. One of the most studied conditions is ventilator-associated pneumonia (VAP), a severe nosocomial infection that typically occurs in patients undergoing prolonged invasive mechanical ventilation. VAP is associated with poor prognosis and high mortality, largely due to its frequent association with multidrug-resistant pathogens, making timely antibiotic therapy crucial for improving outcomes [38]. Given the need for early and reliable diagnostic tools, researchers initially focused on conventional inflammatory markers such as C-reactive protein (CRP) and procalcitonin (PCT). Although these biomarkers increase during VAP, their diagnostic specificity is limited, as similar elevations occur in other respiratory conditions like ventilator-associated tracheobronchitis, making differential diagnosis challenging [39]. In this context, PSP has emerged as a potential biomarker of interest. However, Ceccato et al. reported that isolated PSP values, even when interpreted independently of CRP and PCT, were not sufficiently accurate to support an early diagnosis of VAP. The study’s limitations, however, warrant cautious interpretation and call for further research to validate these findings [40,41]. Despite these diagnostic limitations, PSP appears to hold significant prognostic value in VAP. As demonstrated by Boeck et al., PSP plasma concentrations measured seven days after disease onset correlate with clinical outcomes. Specifically, levels below 24 ng/mL were associated with a favorable prognosis, whereas values exceeding 177 ng/mL predicted poor outcomes. These data suggest that PSP may be a useful prognostic biomarker in patients with VAP [42]. Remaining within the domain of respiratory infections, Scherr et al. explored the utility of PSP in the context of chronic obstructive pulmonary disease (COPD) exacerbations. Their study demonstrated that elevated PSP levels (>33.9 ng/mL), particularly when combined with clinical features such as purulent sputum, were indicative of bacterial infection. These findings highlight the potential of PSP to support etiological differentiation in COPD exacerbations, aiding in the early identification of bacterial causes and informing targeted treatment strategies [42].

### 3.7. PSP and Intra-Abdominal Infections

Intra-abdominal infections (IAIs), particularly peritonitis, represent serious and potentially life-threatening complications of abdominal surgery, frequently requiring admission to intensive care units (ICUs). These infections are often caused by multidrug-resistant organisms, and they are associated with high morbidity and mortality rates [43,44,45]. Timely diagnosis and prognostication are essential for improving clinical outcomes, and pancreatic stone protein (PSP) has emerged as a promising biomarker in this context. Ventura et al. described a compelling clinical case in which an unexpected rise in PSP plasma concentration enabled the early detection of peritonitis in a postsurgical patient without overt abdominal signs. This prompted immediate imaging and intervention, ultimately allowing for the timely management of incipient septic shock [46]. More broadly, PSP has been investigated as a prognostic marker in intra-abdominal infections. Michailides et al. demonstrated that high serum levels of PSP were associated with complicated IAIs, sepsis, an increased need for intensive care, and the greater use of antibiotics and vasopressors. Importantly, PSP levels rose earlier than conventional markers such as C-reactive protein (CRP) and procalcitonin (PCT), and they outperformed ferritin and fibrinogen in predicting clinical deterioration [47]. Gukasjan et al. further confirmed these findings, showing that PSP was superior to CRP, PCT, and interleukin-6 (IL-6) in predicting both the severity of peritonitis and the risk of mortality [48]. Their study highlighted PSP’s utility in guiding early therapeutic decisions and risk stratification. In line with these observations, Eggimann et al. have emphasized PSP’s added value in the early identification of infectious complications in surgical ICU patients, suggesting that it can complement existing clinical scores such as SOFA or APACHE II [14]. In a multicentric cohort, Que et al. reported that combining PSP with other biomarkers improved the predictive accuracy for septic complications and adverse outcomes among patients undergoing abdominal surgery [21]. Additional support for the role of PSP in abdominal infections comes from Prazak et al., who, in a systematic review and patient-level meta-analysis, found PSP to be one of the most accurate biomarkers for infection detection in hospitalized patients, particularly in surgical and critical care settings [15]. Taken together, these findings support the role of PSP not only in the early detection of intra-abdominal infections but also as a valuable prognostic and severity stratification tool, potentially improving the timeliness and appropriateness of clinical interventions.

### 3.8. PSP and Surgery

The potential utility of pancreatic stone protein (PSP) as a biomarker has also been explored in surgical patients. Filippidis et al. investigated PSP levels in patients admitted to the intensive care unit (ICU) following complicated abdominal surgeries, including ruptured anastomoses and bowel perforations. In this high-risk group, the early, non-invasive identification of sepsis is critical due to the frequent occurrence of infectious complications. Their findings support PSP as a valuable biomarker for the early detection of sepsis in these patients, facilitating timely intervention [49]. Similarly, Klein et al. examined the role of PSP in patients undergoing cardiac surgery, with a particular focus on procedures involving cardiopulmonary bypass, which elicit a pronounced systemic inflammatory response [50]. Traditional biomarkers of sepsis, such as C-reactive protein (CRP) and procalcitonin (PCT), are typically elevated postoperatively due to surgical trauma, limiting their specificity for infection diagnosis. In contrast, the study demonstrated that PSP plasma levels remain unaffected by tissue injury caused by cardiac surgery, including those performed with extracorporeal circulation. This characteristic suggests that PSP may serve as a reliable biomarker for the early detection of postoperative infectious complications in cardiac surgery patients. Table 1 resumes diagnostic and prognostic performance of pancreatic stone protein (PSP) compared to commonly used biomarkers across selected clinical conditions.

**Table 1:** Diagnostic and prognostic performance of pancreatic stone protein (PSP) compared to commonly used biomarkers across selected clinical conditions.

Study (Year)	Clinical Condition	Sample Type	Method	Participants (Cases/ Controls)	Biomarker(s) Evaluated	Timing of Elevation	AUC (Statistical Model)	Prognostic Value	Key Findings
Pugin et al. [16]	Sepsis	Plasma	POC Immunoassay0	243/NA	PSP, CRP, PCT	PSP: day 5; CRP/PCT: day 2–3	PSP: 0.87 (ROC); CRP: 0.75; PCT: 0.78	High correlation with severity and organ failure	PSP rises earlier; combination improves accuracy
Permana et al. [17]	Sepsis	/	ELISA	258/NA	PSP, CRP, PCT	Pre-symptomatic	PSP: 0.89 (ROC); CRP: 0.78; PCT: 0.81	PSP associated with mortality	Early elevation aids risk stratification
Que et al. [20]	Septic shock	Plasma	Elisa	104/NA	PSP, CRP, PCT	Not reported	PSP: 0.85 (ROC); CRP: 0.76; PCT: 0.79	PSP only reliable mortality predictor	Stronger correlation with outcome
Melegari et al. [35] (2023)	COVID-19	Plasma	POC Immunoassay	21/NA	PSP, CRP, PCT	Early phase of symptoms	PSP: 0.83 (ROC); CRP: 0.79; PCT: 0.81	Higher PSP = increased mortality	PSP correlates with severity
Van Singer et al. [36] (2021)	COVID-19	Plasma	POC Immunoassay	141/NA	PSP, CRP, PCT	Early after admission	PSP: 0.69 (ROC); CRP: 0.67; PCT: 0.69	Early mortality predictor	PSP improves CRB-65 score (AUC 0.95)
Scherr et al. [42]	COPD exacerbation (CAP)	Serum	ELISA	39/NA	PSP	On admission	Not reported	Not applicable	PSP > 33.9 ng/mL = bacterial CAP
Ventura et al. [46]	Intra- abdominal infection	Plasma	POC Immunoassay	42/NA	PSP	Early postsurgical	PSP: 0.89 (ROC)	Predicts septic complications	PSP rise precedes peritonitis symptoms

## 4. Other Applications of PSP

### 4.1. PSP in Renal Diseases

Hayakawa et al. observed an interesting increase in serum PSP levels even in patients with chronic renal failure, with a significant increase especially in hemodialysis patients, regardless of the presence or absence of pancreatic disease [13]. In addition to this, the presence was also observed in urine, which was also confirmed by the research team of Verdier et al. The protein has been localized at the level of the proximal tubule, and its role is said to overlap with that played at the pancreatic level; in particular, it is supposed to inhibit the formation of calcium carbonate crystals, playing a protective role against the formation of kidney stones [51]. Its presence has been found not only in the serum but also in the urine of healthy patients and patients with kidney stones.

### 4.2. PSP in Type 1 Diabetes

Several important studies have investigated the role of pancreatic stone protein (PSP) in type 1 diabetes, an autoimmune disease characterized by the extensive loss of pancreatic beta cells—specialized islet cells responsible for insulin production and, consequently, glucose metabolism. The disease is marked by the development of autoantibodies targeting various surface antigens on beta cells, which triggers an immune response leading to their destruction. This process results in the inability to produce insulin, impairing glucose clearance from the bloodstream and leading to persistent hyperglycemia [52]. A distinctive feature of beta cells is their limited regenerative capacity, which contributes to the irreversible progression of type 1 diabetes once the autoimmune destruction has begun [53]. As a result, strategies aimed at preserving or restoring beta-cell mass have become central to disease prevention and treatment [54,55]. Subsequent studies identified a novel gene expressed by pancreatic beta cells, believed to play a critical role in promoting the regeneration of damaged cells, thereby improving glycemic control. However, in patients with type 1 diabetes, specific autoantibodies against the protein product of this gene have been observed, potentially interfering with beta-cell regeneration and accelerating disease progression [56,57]. This protein was initially named Reg protein, although it has since been shown to be structurally identical to PSP [58]. This led to the hypothesis that PSP may itself be a target antigen and that antibodies directed against it could be involved in beta-cell destruction and impaired regeneration. Supporting this hypothesis, Astorri et al. reported elevated serum levels of PSP in patients with both newly diagnosed and long-standing diabetes (type 1 and type 2), while anti-PSP antibodies were detected exclusively in individuals with type 1 diabetes. Their findings suggest that PSP could potentially serve as a biomarker for monitoring autoimmunity and beta-cell destruction in type 1 diabetes [59].

### 4.3. PSP in Type 2 Diabetes

Type 2 diabetes mellitus (T2DM) is characterized by progressive pancreatic beta-cell dysfunction and the development of insulin resistance in peripheral tissues [60]. In the early stages of the disease, increased activity of the remaining functional beta cells may temporarily compensate for insulin resistance [61]. The diagnosis of T2DM is typically established by a fasting plasma glucose level > 126 mg/dL, a hemoglobin A1c > 6.5%, or a plasma glucose level > 200 mg/dL following an oral glucose tolerance test [62].

T2DM has a profound impact on patients’ quality of life, long-term survival, and healthcare resources [63]. It is also a major contributor to microvascular and macrovascular complications, including nephropathy, neuropathy, retinopathy, and cardiovascular disease, all of which significantly worsen patient outcomes [64].

In this context, Yang et al. investigated the role of pancreatic stone protein (PSP) in the onset and progression of T2DM, as well as in the development of diabetes-related complications [65]. Their study revealed that PSP levels were already elevated in individuals at high risk for developing T2DM, particularly in those with impaired glucose tolerance—a prediabetic state defined by post-load glucose levels between 140 and 199 mg/dL. PSP concentrations were further elevated in patients with an established diagnosis of T2DM, with values positively correlated with disease duration and the presence of vascular complications.

### 4.4. PSP in Gastrointestinal Cancer

The potential role of the *Reg/PSP* gene in oncological diseases was first explored by Watanabe et al., who conducted pivotal studies on its expression in both non-pancreatic tissues and various cancer types [66]. They identified low-level gene expression in healthy tissues such as the kidney and stomach but markedly higher expression in gastrointestinal tumors, particularly colorectal and gastric cancers. Hayashi et al. further supported the involvement of Reg/PSP as a potential predictive marker for chemo- and radiosensitivity in esophageal squamous cell carcinoma. Their study demonstrated that cell lines expressing the gene were more responsive to anticancer treatments and associated with improved survival and prognosis compared to non-expressing lines [67].

Several studies have also focused on gastric cancer. Fukui et al. successfully isolated the protein from various gastric epithelial cells in rats, especially enterochromaffin-like cells, and demonstrated that gastrin exposure upregulated PSP expression, inducing mutagenic changes [68]. These results were later confirmed in human cells by Higham et al., who showed that gastrin induced Reg/PSP expression, promoting cell proliferation and mutagenesis. Interestingly, mutations in the *Reg/PSP* gene that blocked protein synthesis were found to exert a protective effect against gastric cancer [69]. Sekikawa et al. showed that gastric tumors with Reg/PSP overexpression were more invasive, mitotically active, and associated with worse outcomes. They also found that cytokines such as interleukin-6 (IL-6) and interferon-gamma (IFN-γ) could stimulate Reg/PSP expression, mimicking the effect of gastrin [70]. Notably, anti-Reg/PSP antibodies were able to inhibit this process, suggesting a potential therapeutic target. Yamagishi et al. added that elevated Reg/PSP expression correlates with poor prognosis in gastric cancer, further establishing its prognostic value [71]. In support of this, Kuniyasu et al. demonstrated Reg/PSP overexpression in peritoneal metastases of gastric carcinoma and in peritoneal lavage fluid, associating these findings with an increased risk of peritoneal carcinomatosis. Elevated PSP levels in lavage fluid may therefore serve as a non-invasive marker for metastatic spread [72].

The role of Reg/PSP has also been examined in colorectal cancer. Bernard-Perrone et al. showed that Reg/PSP enhances cell proliferation and inhibits differentiation in vitro, while its suppression promotes differentiation and reduces mitosis. They also proposed a role in cell junction regulation [73]. Zenilman et al. observed increased protein expression both in cancer cells and adjacent normal epithelium, particularly at the transition zone, suggesting that it may serve as a biomarker for early neoplastic transformation [74]. Elevated Reg/PSP levels in peritoneal carcinomatosis cells were also described by Astrosini et al., who proposed their use as a marker for metastatic potential [75]. Furthermore, Macadam et al. linked high Reg/PSP expression—alone or with high pancreatitis-associated protein (PAP) levels—to increased mortality, even in non-metastatic patients [76]. The protein has also been studied in less common gastrointestinal tumors. Li et al. found high PSP expression in various neuroendocrine tumors—including those of the pancreas, stomach, and colorectum—as well as in pheochromocytoma and medullary thyroid carcinoma, but not in tumors from other locations [77]. Harada et al. investigated intraductal cholangiocarcinoma, showing that PSP expression increased progressively across hyperplasia, dysplasia, and overt cancer but remained absent in healthy tissue [78]. In hepatocellular carcinoma, Yuan et al. observed that high PAP expression was associated with low-grade tumors and favorable prognosis, whereas Reg/PSP overexpression—often accompanied by p53 mutations—was linked to high-grade tumors and worse outcomes [79] In contrast, in gallbladder carcinoma, Tamura et al. found that high PSP levels were associated with malignant transformation via intestinal metaplasia but paradoxically correlated with better survival after surgery, suggesting context-dependent prognostic value [80]. Finally, in pancreatic cancer, Zhou et al. confirmed the mitogenic role of Reg/PSP in both in vitro and in vivo models, noting its association with locally aggressive and invasive forms. They also proposed the inclusion of Reg/PSP among the most promising diagnostic biomarkers for pancreatic carcinoma [81,82].

### 4.5. PSP in Other Forms of Cancer

Unlike gastrointestinal malignancies, the role of pancreatic stone protein (PSP) in other types of cancer has been investigated only in a limited number of studies. In their attempt to identify novel biomarkers for metastatic prostate cancer, Gu et al. found a strong correlation between elevated PSP levels and advanced, hormone-refractory disease, suggesting its potential use in identifying aggressive prostate cancer phenotypes [83]. Similarly, Wang et al. explored the expression of PSP in cerebral gliomas, reporting that higher levels of the protein were associated with poorer survival rates. Their findings support the idea that PSP may serve as an unfavorable prognostic marker in gliomas and may be linked to increased risk of disease progression [84].

Beyond its role in solid tumors, PSP has also been evaluated as a potential biomarker in cancer-associated complications, particularly febrile neutropenia, one of the most common and serious adverse effects of chemotherapy [85]. These patients often lack the classic signs and symptoms of infection, with fever being the only presenting feature, and due to profound immunosuppression, they are at high risk for severe infections, prolonged hospitalization, and elevated mortality [38,85]. Therefore, identifying biomarkers capable of enabling the early and non-invasive detection of infections in this fragile population is of critical importance for guiding timely and appropriate treatment [86,87].

In this context, García de Guadiana-Romualdo et al. assessed the potential diagnostic utility of PSP in comparison to more traditional markers such as procalcitonin (PCT) [88]. Their study showed a significant correlation between elevated PSP levels and the occurrence of infection in patients with febrile neutropenia following chemotherapy. However, they did not find PSP to be superior to PCT. As a result, they concluded that while PSP may have diagnostic potential, it does not currently outperform existing markers, and thus PCT remains the preferred biomarker in clinical practice.

### 4.6. PSP in Liver Failure

Another potential area of application for pancreatic stone protein (PSP) is in the evaluation and diagnosis of liver diseases, particularly in cases of acute liver failure (ALF). ALF is a rare but life-threatening condition that develops suddenly in previously healthy individuals and is clinically defined by the triad of coagulopathy, hepatic encephalopathy, and acute liver injury. While its causes vary, including viral infections, drug-induced hepatotoxicity, and autoimmune diseases; the condition is marked by rapid clinical deterioration and high mortality [89].

One of the major contributors to poor outcomes in both ALF and acute-on-chronic liver failure (ACLF) is the onset of secondary infections, which further compromise liver function and increase the risk of multiorgan failure. Therefore, the early and accurate identification of infections in these patients is crucial for initiating timely and targeted antimicrobial therapy. However, standard infection biomarkers such as C-reactive protein (CRP) and procalcitonin (PCT) have limited reliability in this setting. CRP levels may be elevated in patients with cirrhosis regardless of the presence of infection, and paradoxically, they may be lower in advanced liver disease due to impaired hepatic protein synthesis [90]. Similarly, PCT is often more closely associated with hepatocellular injury and necrosis than with infection itself, reducing its diagnostic specificity in liver failure [91].

In light of these limitations, Lopes et al. investigated the potential role of PSP as an alternative biomarker in patients with ALF and ACLF. Their findings showed a general elevation of PSP levels in patients with liver injury—both in acute and chronic decompensated forms. However, no significant difference in PSP concentrations was observed between infected and non-infected individuals, indicating limited utility for diagnostic discrimination.

Interestingly, among patients with infection, those with higher PSP levels exhibited significantly increased mortality compared to those with lower levels. Based on this observation, the authors proposed a prognostic role for PSP in liver failure, suggesting that elevated PSP concentrations in infected patients with ALF or ACLF may help identify individuals at higher risk of death, even if PSP cannot distinguish infection status per se [92,93].

#### Strengths and Limitations

This review offers a comprehensive synthesis of the current evidence on pancreatic stone protein (PSP), highlighting its emerging role as a biomarker across a wide spectrum of clinical conditions from sepsis and intra-abdominal infections to oncological and metabolic diseases. One of the main strengths of this work is the breadth of its scope, which encompasses both diagnostic and prognostic aspects of PSP, as well as its underlying biomolecular characteristics. The use of structured literature search methods and a clinically oriented narrative framework allows for a nuanced interpretation of the available findings, with attention to pathophysiological plausibility and translational relevance.

Moreover, the review includes recent studies that explore the performance of PSP in comparison to traditional biomarkers such as CRP and procalcitonin, offering a timely appraisal of its added value and limitations in real-world clinical settings. The inclusion of heterogeneous clinical contexts—from intensive care and oncology to chronic diseases—reflects the protein’s potential as a systemic marker of inflammation, stress response, and tissue damage.

However, several limitations must be acknowledged. First, as a narrative review, this work does not follow a systematic review methodology and, therefore, may be subject to selection bias and publication bias. Although efforts were made to include recent and high-quality studies, the inclusion criteria were not strictly predefined, and some relevant studies may have been inadvertently excluded. Second, the heterogeneity of study designs, patient populations, and PSP assay methods across the literature makes direct comparisons difficult and limits the ability to draw definitive conclusions regarding diagnostic thresholds or clinical utility. In particular, variations in PSP cut-off values and assay platforms hinder the generalizability of results.

Additionally, many of the studies discussed are observational or exploratory in nature, and few are capable of assessing clinical outcomes in a prospective manner. The lack of standardized protocols for PSP measurement and the relatively limited number of randomized controlled trials represent major gaps in the current evidence base. Future research should aim to validate PSP’s diagnostic and prognostic performance through large, multicenter trials using harmonized methodologies.

## 5. Conclusions

Pancreatic stone protein (PSP) is emerging as a promising biomarker in various clinical contexts, with potential applications in both diagnostic and prognostic domains. Its early rise in bloodstream concentrations, especially in response to infectious and inflammatory stimuli, makes it an attractive candidate for timely clinical decision-making, particularly with critically ill patients and those facing complex conditions such as sepsis, intra-abdominal infections, and febrile neutropenia.

Despite encouraging evidence, PSP is not yet ready to replace established biomarkers such as CRP or procalcitonin in routine practice. Rather, its use should currently be considered complementary, particularly in situations where standard markers provide inconclusive results or are affected by underlying conditions like liver failure or immunosuppression. Furthermore, the potential role of PSP in predicting disease severity and mortality may prove especially useful in triaging patients at higher risk, thereby guiding resource allocation and therapeutic strategies.

To ensure its pragmatic implementation in clinical workflows, future studies should focus on validating PSP in large, multicenter cohorts using standardized assay methods and should also explore its integration into multimarker algorithms and clinical prediction models. Only through such validation can PSP move from experimental use toward evidence-based incorporation into clinical guidelines.

## Data Availability

Not applicable.

## Appendix A

To provide a clearer biological context for the clinical applications of PSP, we present a structured overview of its biomolecular features, based on data from UniProt (P05451) and The Human Protein Atlas. Protein name and taxonomy: pancreatic stone protein (PSP), also known as regenerating islet-derived protein 1-alpha (REG1A), is a secreted protein encoded by the gene in humans (Homo sapiens). It is a member of the regenerating gene (Reg) family, originally identified in association with pancreatic calculi and later implicated in tissue regeneration, inflammation, and tumorigenesis. UniProt ID: P05451. Gene name: REG1A. Organism: Homo sapiens (Human). Alternative names: Lithostathine-1-alpha, PSP, Islet cells regenerating protein I-alpha.

Isoforms and variants: PSP is translated as a single-chain precursor to 166 amino acids, with a molecular weight of approximately 18.3 kDa. The protein includes a signal peptide (residues 1–22) that is cleaved during secretion. No alternative isoforms have been reported for REG1A, although polymorphisms affecting its expression have been described. Length: 166 amino acids. Signal peptide: residues 1–22. Mature protein: residues 23–166. Post-translational modifications: N-glycosylation at Asn43; cleavage of signal peptide. Tissue and cellular expression profile: According to The Human Protein Atlas and transcriptomic data, PSP/expression is highly enriched in the pancreas, especially in acinar cells; it is moderately expressed in the gastrointestinal tract, including the small intestine and stomach. Also detected in kidney proximal tubules and, under pathological conditions, in the liver, the lungs, and immune cells (e.g., neutrophils). Under stress or inflammatory stimuli, its expression can be upregulated within non-pancreatic tissues, reflecting its role in injury response and immune modulation. Functional domains and molecular roles: the PSP protein contains a C-type lectin-like domain (CTLD), which mediates calcium binding and may play a role in recognition of glycan structures. The protein is thought to function in the following:

- The inhibition of calcium carbonate crystallization, particularly in the pancreas and kidney;
- The promotion of epithelial regeneration, through anti-apoptotic and mitogenic effects;
- The modulation of innate immune responses, including neutrophil activation and migration;
- Involvement in tumorigenesis, via pro-proliferative signaling in certain cancer types;
- Emerging data suggest that PSP also exerts cytokine-like activity, although a specific receptor has not yet been identified.

## Appendix B

### Appendix B.1. Scale for the Assessment of Narrative Review Articles (SANRA)

#### Appendix B.1.1. Justification of the Article’s Importance for the Readership

This review highlights the clinical relevance of pancreatic stone protein (PSP), a biomarker with multiple emerging applications across a range of medical conditions. It aims to provide clinicians and researchers with a concise yet comprehensive reference concerning its potential diagnostic and prognostic roles.

#### Appendix B.1.2. Statement of Concrete Aims or Formulation of Questions

The objective of this narrative review was to summarize and critically assess the available evidence regarding the clinical utility of PSP. The review was designed to serve as a practical tool for healthcare professionals seeking to understand where and how PSP can be applied in routine and specialized settings.

#### Appendix B.1.3. Description of the Literature Search

The literature search was conducted using PubMed^®^ as the primary database. Boolean operators (and, or, not) were used to refine the search strategy. The following keywords were employed: “Pancreatic Stone Protein”, “Pancreatitis”, “Renal Disease”, “Diabetes”, “Cancer”, “Gastrointestinal cancer”, “Infections”, “Pneumoniae”, “Sepsis”, “Burns”, and “Surgery”. Relevant studies were selected based on their alignment with the objectives of the review and their contribution to understanding the clinical role of PSP.

#### Appendix B.1.4. Referencing

The review includes a selection of high-impact and representative articles from the current literature. These references were chosen based on methodological rigor, relevance to the clinical applications of PSP, and contributions to the scientific understanding of the biomarker.

#### Appendix B.1.5. Scientific Reasoning

The review was grounded in the interpretation of clinical studies investigating PSP. Emphasis was placed on evidence that elucidates the biomarker’s behavior in different pathophysiological conditions, including comparisons with standard biomarkers when available.
